# Self-Organizing Maps-based ocean currents forecasting system

**DOI:** 10.1038/srep22924

**Published:** 2016-03-16

**Authors:** Ivica Vilibić, Jadranka Šepić, Hrvoje Mihanović, Hrvoje Kalinić, Simone Cosoli, Ivica Janeković, Nedjeljka Žagar, Blaž Jesenko, Martina Tudor, Vlado Dadić, Damir Ivanković

**Affiliations:** 1 Institute of Oceanography and Fisheries, Šetalište I. Meštrovića 63, 21000 Split, Croatia; 2 University of Split, Faculty of Science, Teslina 12, 21000 Split, Croatia; 3 Istituto Nazionale di Oceanografia e di Geofisica Sperimentale, Borgo Grotta Gigante 42/c, 34010 Sgonico, Trieste, Italy; 4 University of Western Australia, School of Civil, Environmental and Mining Engineering, 35 Stirling Highway, Crawley WA 6009, Australia; 5 Ruđer Bošković Institute, Bijenička cesta 54, 10000 Zagreb, Croatia; 6 Faculty of Mathematics and Physics, University of Ljubljana, Jadranska 19, 1000 Ljubljana, Slovenia; 7 Meteorological and Hydrological Service, Grič 3, 10000 Zagreb, Croatia

## Abstract

An ocean surface currents forecasting system, based on a Self-Organizing Maps (SOM) neural network algorithm, high-frequency (HF) ocean radar measurements and numerical weather prediction (NWP) products, has been developed for a coastal area of the northern Adriatic and compared with operational ROMS-derived surface currents. The two systems differ significantly in architecture and algorithms, being based on either unsupervised learning techniques or ocean physics. To compare performance of the two methods, their forecasting skills were tested on independent datasets. The SOM-based forecasting system has a slightly better forecasting skill, especially during strong wind conditions, with potential for further improvement when data sets of higher quality and longer duration are used for training.

## Introduction

Ocean and environmental forecasting at regional or basin-wide scales^1^ can be done in different ways, depending on the processes to be investigated, and the time and spatial scales of interest^2^. The most common approach at regional scales and for short-term forecasts (up to a week), is to use regional ocean models driven by mesoscale atmospheric model outputs. Forcing is usually carried out through one-way coupling, using multiple nesting techniques and with lateral boundary conditions coming from larger or global models^3^. Other approaches, developed in recent decades, include multi-ensemble methodologies, different data assimilation techniques^4^, hybrid modeling^5^ and neural network modeling^6^. The latter is often based on the Self-Organizing Maps (SOM) algorithm^7^.

Since the 2000s the Self-Organizing Maps method has been introduced more extensively in atmospheric and ocean sciences^8^, above all for mapping of: climate and atmospheric processes^9^, remote sensing data^10^, and ocean modeling data^11^. The method performs best on comprehensive and long time series, and it is particularly suitable for mapping surface current fields from moored current-meters and high-frequency (HF) radars in coastal areas^12^,^13^,^14^. However, a SOM-based operational forecasting method has not been implemented yet in any operational oceanography network, in spite of the fact that a similar approach, in combination with other techniques, is already used for instance in energy systems^15^, and hydrology^16^. This paper documents the architecture of a SOM-based forecasting system, specifically designed for surface currents, and its evaluation vs. more conventional ocean forecasting tools (specifically, the ROMS ocean model). The system is based on numerical weather prediction (NWP) from the atmospheric model ALADIN/HR, on HF radar data and on an unsupervised SOM training. Skill assessment for such system is done and relevant metrics are estimated. System performance is compared to the existing operational ocean forecasting system based on the ROMS ocean model, which is coupled with the same ALADIN/HR NWP model.

### The SOM-based forecasting system and the operational ROMS setup

**Figure 1:**
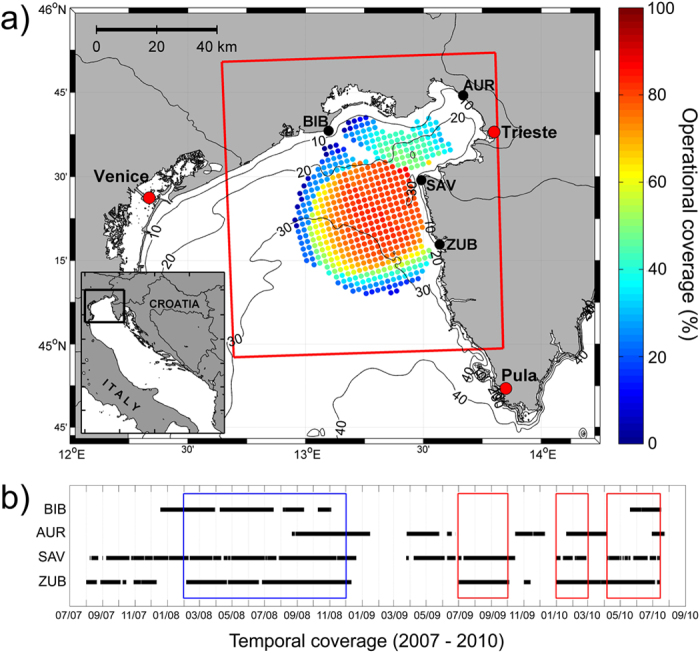
(**a**) The domain of the SOM-based operational forecasting system in the northern Adriatic with marked HF radar stations (BIB – Bibione, AUR – Aurisina, SAV – Savudrija, ZUB – Zub). Operational coverage of hourly surface currents over the predefined Cartesian grid during testing periods is given in percent, while spatial coverage of the Aladin/HR model used for training and forecasting is denoted by the red rectangle. Operational coverage at the same Cartesian grid during testing period was higher than 60%. (**b**) HF radar operability between 2007 and 2010 with marked training (blue rectangle) and testing (red rectangles) periods. The figure has been created using MATLAB (www.mathworks.com) and CorelDRAW (www.corel.com) software.

**Figure 2:**
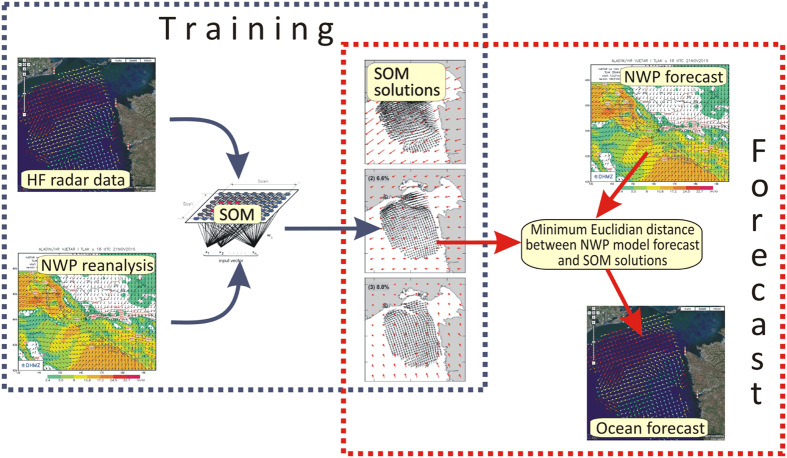
The architecture of the SOM-based operational forecasting system. The figure has been created using CorelDRAW (www.corel.com) software.

The SOM-based operational system was developed with the purpose of providing surface current forecasts (up to 72 h) in the northern Adriatic Sea (Fig. 1) as part of the NEURAL project (http://jadran.izor.hr/neural). The system is based on the assumption that local wind is the major driver of near-surface circulation in the region. The temporal extent of the ocean current forecast window is dictated by the available NWP weather forecast, which extends to 72 hours. The system is based on training and forecast phases (Fig. 2). The main goal of the training phase is to find a robust link between ocean surface currents and surface wind fields. In order to do so, the following steps are undertaken: (1) surface currents in the area are measured using HF radars during a prolonged time interval; (2) sea-surface wind fields are obtained from a high-resolution mesoscale atmospheric model; (3) the characteristic surface current patterns are extracted from HF radar data using the SOM method; (4) joint surface current and wind patterns are extracted from wind and surface currents using the SOM method; (5) then, optimal SOM parameters are estimated and the method applicability is tested on available data. The forecasting phase is based on: (1) forecasting surface winds with a high resolution mesoscale atmospheric model; (2) recognizing characteristic SOM patterns (as determined during the training phase) in the surface wind forecast by searching for the minimum Euclidian distance between characteristic patterns and the NWP-forecasted surface winds; and (3) assigning the corresponding surface current pattern as the surface current forecast.

For this study HF radar data were acquired from four SeaSonde radar systems, located at Bibione (BIB), Aurisina (AUR), Savudrija (SAV) and Zub (ZUB) (Fig. 1), that were operational from 2007 to 2010. Radars worked at 25 MHz, were set up with 512 fft points, 2 Hz sampling rate and a bandwidth of 100 kHz, corresponding to a radial resolution of 1.5 km in range, and 2.3 cm/s in radial velocity. Angular resolution was 5°, and the maximum operating range was 50 km. Hourly surface currents were estimated on a predefined 2 km × 2 km Cartesian grid (Fig. 1) by applying a least-squares algorithm^17^. Radial currents were quality-checked to remove Doppler lines that were either poorly constrained by signal-to-noise (SNR) ratio or had insufficient spectral quality factor^18^. Total vectors were also checked for spikes^19^ and spatial gaps were filled using a Gaussian-weighted spatial interpolation. The period with the best temporal coverage (operational coverage from 60% at the distant points up to 100% in the center of the radar domain) and having the highest quality of data was the period between February and November 2008, so this dataset was used for the SOM training phase.

High-resolution surface wind fields (2 km × 2 km) obtained with the ALADIN (Aire Limitée Adaptation Dynamique Développement Inter National) mesoscale NWP model^20^, have been used both in training (jointly with surface currents data in the period February-November 2008) and forecast phase. The ALADIN/HR version of the NWP model which has been used for the Adriatic Sea has 37 levels in the vertical, 2 km resolution in horizontal, and a full set of physics parameterizations, including prognostic TKE (turbulent kinetic energy), microphysics and a prognostic convection scheme^21^. Hourly files were used for the training.

Both surface currents and surface winds were preprocessed with a 33-h low-pass filter, to remove tides and high-frequency atmospheric and ocean processes from the data. Then, current data in the training period (February-November 2008) were introduced to the 4 × 5 SOM of the sheet type and 20 characteristic surface current patterns were mapped. Next, a joint set of surface currents and surface winds was introduced, again to the 4 × 5 SOM of the sheet type and another 20 characteristic joint surface currents surface wind patterns have been obtained. Complex correlations coefficients with values from 0.880 to 0.999 between SOM patterns based on surface current data only and patterns based on the joint surface current and surface wind data were estimated^22^. High correlation between these two sets of SOM patterns is a prerequisite for creating SOM ocean surface currents based on wind forecasts.

Following the testing phase, three distinct periods, 1: July-September 2009, 2: January-February 2010, and 3: April-July 2010, were used for testing reliability of the SOM-based operational forecast (Fig. 1). BIB and AUR stations were largely out of operation during the three testing periods, therefore operational coverage was as low as 20% at some grid points (Fig. 1), much lower than operational coverage during the training phase (at least 60% at the same Cartesian grid). A forecast of surface currents was verified on measured data and standard metrics (RMSE) were computed. The 4 × 5 SOM matrix was chosen for the forecasting system, resulting in 20 patterns of forecast surface currents, as this choice provides the minimum root-mean-square error (RMSE) between the measured and forecast surface current patterns when compared to other tested sets of SOM matrices (from 2 × 2 to 16 × 12 SOM).

The parallel ocean forecasting system, based on the ROMS ocean model (Regional Ocean Modeling System^23^), is operational in the Adriatic Sea since 2008. The Adriatic ROMS model has a 2 × 2 km horizontal resolution, 20 sigma layers in the vertical; it is forced by the ALADIN/HR NWP fields at the surface. Lateral boundary conditions come from the larger AREG model^24^ and river climatology^25^. Further details of the modeling system can be found in this paper^25^. Modeled ROMS surface current data filtered with a 33-h low pass filter were recomputed over the HF radar grid for the three testing periods to allow comparison with the SOM-based surface current forecast.

### Evaluation of the SOM-based forecast

Average RMSE from the SOM-based forecast is 9.4 cm/s for period 1, 7.7 cm/s for period 2 and 8.4 cm/s for period 3. The best metrics are estimated during the winter period (period 2), while they are much larger during summer and spring periods (periods 1 and 3, respectively). The respective ROMS-based metrics have a similar level of quality, with slightly higher RMSE values during summer (period 1: 10.0 cm/s) and spring (period 3: 9.8 cm/s) and lower during winter period (period 2: 7.5 cm/s). Averaged over all testing periods, the ROMS-based forecast is 8.7% worse than the SOM-based forecast.

**Figure 3:**
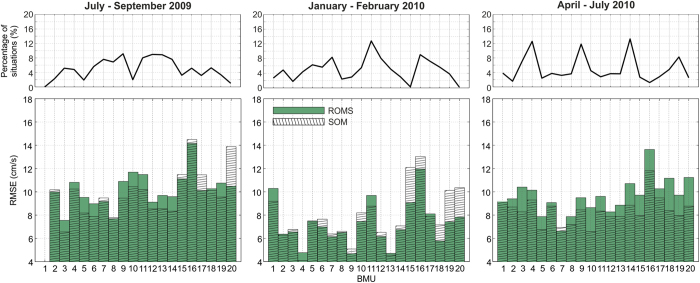
Distribution of root-mean-square error (RMSE) between SOM- and ROMS-derived forecast of surface currents and the respective measurements per BMU and for three testing periods. Percentage of situations ascribed to a particular BMU is also shown. The figure has been created using MATLAB (www.mathworks.com) software.

**Figure 4:**
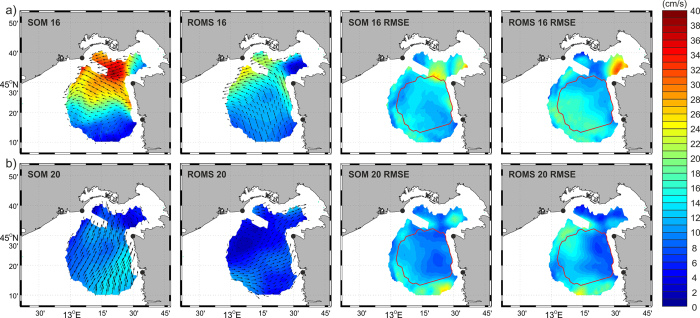
Surface currents obtained by the SOM-based forecasting system, the respective average surface currents obtained by ROMS forecasting system, and the RMSE between SOM-based and ROMS-based forecast of surface currents and the measurements, computed for (**a**) BMU16 (associated with strong bora wind), and (**b**) BMU20 (associated with sirocco wind). Encircled area includes grid points with at least 60% operational coverage during the testing period. The figure has been created using MATLAB (www.mathworks.com) software.

We have further estimated both ROMS and SOM forecast system skill changes over different SOM solutions (Fig. 3). For any given SOM pattern we computed: (1) RMSE between the current field characteristic for that pattern and the surface current field obtained from measurements; (2) RMSE between ROMS currents and the surface current field obtained from measurements; both (1) and (2) were computed for those times for which a particular SOM pattern was forecasted. The pattern BMU16, associated to strong bora wind (Fig. 4), has the largest RMSE values. During wintertime (period 2), this pattern appears more frequently than during other investigated periods (9% in period 2, 1.5% in period 1 and 5% during period 3) and is slightly better forecast by the ROMS than by the SOM forecast system (Fig. 3). On the other hand, it is better forecast by the SOM during periods 1 and 3, but its representation in all patterns is smaller (1.5 and 5%, respectively). Another important extreme wind pattern, BMU20, is associated with the sirocco wind blowing from the south (Fig. 4). This pattern has a much lower frequency (up to 3%) during testing periods (Fig. 3). Its SOM-based forecast also provides larger errors than the ROMS forecast during periods 1 and 2, but smaller errors in period 3 when the pattern BMU20 is slightly more frequent. The overall shape of the RMSE distribution over the BMUs (Fig. 3) shows more resemblance between periods 1 and 3, which are characterized by vertical stratification in the sea, than in period 2, when the sea is usually homogenized^26^. Also, RMSE is much smaller during period 2 than during periods 1 and 3. Therefore, baroclinicity introduces additional error to both SOM- and ROMS-based forecasts.

SOM and ROMS forecast for BMU 16 and BMU 20 are analyzed in more detail. The BMU16 is a result of extreme bora conditions. The bora wind is strongly horizontally variable over a kilometer spatial scale in the northern Adriatic^27^, and it might not be properly represented by the 2-km resolution ALADIN/HR mesoscale model. Mesoscale atmospheric models tend to largely underestimate energies at high frequencies^28^, thus implying that higher resolution is needed in the model to fully describe the bora wind dynamics.

For that reason surface currents forecast by the SOM-based operational system – which are based on real measurements – are more intense and reliable during extreme events than surface currents forecast by ROMS. As seen in Fig. 4, the SOM-forecasted surface currents are much stronger (30–40%) than ROMS-forecasted values. Also, ROMS-forecasted fields corresponding to BMU16 wind conditions contain a northward current in the southern part of the domain, being a part of the cyclonic circulation known to occur in the area of the northern Adriatic during strong bora forcing^29^. By contrast, the SOM-based forecast has very weak currents in the southern part of the domain associated with the BMU16 pattern, followed by a lower RMSE than the one of the ROMS-based forecast (Fig. 4). Two alternative explanations for the discrepancy are feasible: (i) ALADIN/HR wind forcing during wintertime extreme bora wind creates an artificial strengthening of the cyclonic gyre in the area of the northern Adriatic, frequently reproduced by ocean numerical models which use atmospheric mesoscale models^30^, or (ii) HF radar measurements are of lower quality at the southernmost part of the domain, which is covered by two HF radars only.

The difference between the SOM- and ROMS-based forecasts is also evident during intervals represented by other patterns, like the BMU20 (Fig. 4), which is representative of the second dominant wind in the northern Adriatic, the sirocco^31^. Although currents are not as strong as during strong bora episodes, the SOM-based forecast still gives 20–40% stronger currents than the ROMS-based forecast. In addition, there is a 10–30% clockwise offset in ROMS-forecasted current direction.

The spatial distribution of RMSE during periods corresponding to BMU16 and the BMU20 patterns (Fig. 4) reveals that both SOM- and ROMS-based forecasting systems reproduce mean current field relatively well (small RMSE in area with temporal coverage higher than 60%). On the other hand, very high RMSE values in areas with relatively low temporal coverage reveal that variability of the measurements is much higher than variability of both SOM and ROMS forecasts and that this variability is not adequately reproduced by either of the two forecast models.

### Discussion and perspectives

Both SOM- and ROMS-based forecasting systems perform short-term (72 h) forecasting of surface currents in the northern Adriatic with similar skill and quality. However, a limited dataset used for training of the SOM-based forecast, including only 10 months of HF currents in the northern Adriatic, presumably lower the quality of the SOM-based forecast. An increase of the training dataset to several years would likely improve the skill of the forecast, allowing for better and more reliable selection of an optimal number of SOM patterns. Such datasets are now available from HF radar measurements in numerous other regions of the World Ocean^32^, so this approach can be easily tested.

There are several advantages when using SOM-based neural networks for forecasting ocean parameters, in our case surface currents. The first is that the system is based on real data, with a much simpler model learning approach than in other techniques based on real data, such as data assimilation^33^. Another advantage is that, after HF radar time series of sufficient length are collected and a SOM-based forecasting system for an area has been created, HF radars can be redeployed in other coastal areas, potentially allowing for a spatial extension of the SOM-based operational forecasting. Finally, the required time for execution of the SOM-based forecast is much smaller, an order of magnitude, than for forecasts provided by ocean numerical models.

On the other hand, a prerequisite for the proposed forecasting system is a high correlation between predictor and predictand, in our case between wind and surface currents. However, such a high correlation is common for coastal areas^34^, widening application of a SOM-based forecasting system for surface currents at a number of coastal regions where HF radar measurements are available. Another problem is an under-representation of extreme situations in the forecasting system - and forecasting of extremes has much more practical use than forecasting of mean situations - if not trained by a satisfactorily number of BMU patterns. Such a problem may be overcome by a two-step SOM, i.e. by first training it on all measured current fields and then by re-training it only on those current fields which are representative for extreme conditions (e.g., BMU16 and BMU20 in our case). This would downscale the training process through use of a number of BMU solutions instead of one representative for extreme conditions. However, this should be accompanied with an increasing resolution of atmospheric numerical models - enabling them to recognize more variable wind fields. Also, a use of supervised neural network algorithms^35^ with prescribed patterns in predictands might be a solution for improving the skill of the SOM-based forecasting system.

The evaluated SOM-based forecasting system in the northern Adriatic is performed on low-pass (33 h) filtered ocean currents and winds, and therefore it does not account for tides and other processes over hourly timescales, such as inertial oscillations or mesoscale phenomena originating outside of the domain. These processes might be significant in marginal seas (tidal currents are about 10 cm/s in the area^36^). For that reason, we tested both SOM and ROMS-based forecasting systems without preprocessing (low-pass filtering) on the same training and testing dataset, resulting in RMSE increase for about 60% for both forecasting systems compared to the computations with preprocessing. Nevertheless, the RMSE of the SOM forecast is on average about 9.5% smaller than the RMSE of the ROMS forecast. However, the increase of the RMSE in the SOM-based forecasting system might be lowered by removing tides prior to the SOM analyses and re-adding them later to the forecast patterns. Also, a significant increase of the testing dataset might allow for use of much more SOM patterns than used here (20 patterns), which, in the end, might result in more reliable reproduction of all processes appearing in a domain.

The proposed SOM-based forecasting system may be particularly applicable for areas with heavy marine traffic, harbors, touristic areas, etc., where forecasting of surface currents may be essential for preventing and mitigating impacts of marine accidents (e.g., oil spill, rescue at sea, safety of navigation). A similar architecture of the forecasting system could also be appropriate for forecasting different parameters in marine systems (in biogeochemistry, trophic relations, fisheries^37^) which are too complex for “classical” forecasting tools based on numerical models.

## Additional Information

**How to cite this article**: Vilibić, I. *et al.* Self-Organizing Maps-based ocean currents forecasting system. *Sci. Rep.* **6**, 22924; doi: 10.1038/srep22924 (2016).
